# Assessing the Role of Trust in Information Sources, Adoption of Preventive Practices, Volunteering and Degree of Training on Biological Risk Prevention, on Perceived Risk of Infection and Usage of Personal Protective Equipment Among Italian Medical Students During the SARS-CoV-2 Pandemic

**DOI:** 10.3389/fpubh.2021.746387

**Published:** 2021-10-28

**Authors:** Elisa Maietti, Manfredi Greco, Chiara Reno, Flavia Rallo, Davide Trerè, Elena Savoia, Maria Pia Fantini, Lawrence M. Scheier, Davide Gori

**Affiliations:** ^1^Department of Biomedical and Neuromotor Sciences, Alma Mater Studiorum-University of Bologna, Bologna, Italy; ^2^Department of Experimental, Diagnostic and Specialistic Medicine, Alma Mater Studiorum-University of Bologna, Bologna, Italy; ^3^IRCCS Azienda Ospedaliero-Universitaria di Bologna, Bologna, Italy; ^4^Emergency Preparedness Research Evaluation & Practice Program, Harvard T.H. Chan School of Public Health, Boston, MA, United States; ^5^LARS Research Institute, Prevention Strategies, Scottsdale, AZ, United States

**Keywords:** COVID-19, preventive measure, medical student, voluntary service, risk perception, source of information, survey

## Abstract

**Background:** During the initial phase of the COVID-19 pandemic, the University of Bologna Medical School surveyed medical students to learn more about their preparation to confront challenges posed by the pandemic and whether it affects perceptions of viral infection risk. This information could help design risk-reduction interventions with training to mitigate possible viral exposure.

**Method:** A cross-sectional online survey examining students' characteristics, volunteer status, adoption of evidence-based preventive measures, trust in information sources used, infectious disease training, and knowledge of PPE usage in relation to perceived risk of infection from SARS-CoV-2 in daily living, academic, and healthcare activities. A multivariate path model estimated the simultaneous influences of all exogenous factors on perceived risk. A Poisson regression model assessed the same multivariate effects on knowledge of PPE usage.

**Results:** The analysis sample included 537 respondents. Perceived risk of infection was highest in hospital activities. On average, students were able to use only four out of seven types of PPE albeit they adopted most of the evidence-based preventive measures. Adoption of preventive measures was positively associated with perceived risk of COVID infection. Conversely, training on PPE usage and volunteer work were associated with lower perceived risk in healthcare setting and higher PPE knowledge.

**Conclusion:** Implementing early safety-based educational programs remedy students' lack of knowledge in infectious disease prevention and mitigate their risk of infection. Voluntary work should be encouraged with potential benefit for both their continued medical training and strengthening the healthcare system's response to public health emergencies.

## Introduction

Italy was the first European country to report a case of Coronavirus Disease (COVID-19) on February 19, 2020. In the ensuing weeks, there was an exponential increase in the number of cases and deaths due to COVID-19 spreading rapidly to neighboring regions (1) with major disruptions to academic centers of higher learning.

Alma Mater Studiorum–University of Bologna is a major academic institution located in Northern Italy with 87,590 students, including two cohorts enrolled in the Medicine and Surgery program (2). On February 23, 2020, the Dean of the University instructed all faculty, staff, and students to take precautionary measures to prevent the spread of COVID-19. This included suspension of all teaching and training activities and transitioning to online learning.

On March 9, 2020, the Italian government enforced a national lockdown restricting mobility between jurisdictions and forcing closure of non-essential services and educational institutions. By March 11, 2020, 94% of courses at Bologna University were online (3). In keeping with this transition, the medical school suspended all teaching activities and clinical rotations. Separately, self-organized medical students (*n* = 300) created a volunteer group (“A un metro da te” or one meter distance) to help alleviate the workload of healthcare providers delivering care in and out of hospital settings (4). May 2020 witnessed a considerable reduction of COVID-19 cases throughout Italy (5). At that point, the Medical School Board requested information regarding medical students' opinions about the possibility of returning to in-person activities.

In this context, we conducted a survey to collect information regarding medical students' perceptions of the pandemic in relation to their educational activities. The goals of the survey included learning more about sources of COVID-19 information, students' trust in these sources, practices regarding protective measures, perceived risk of contracting COVID-19, and students' knowledge of using personal protective equipment (PPE). In addition, we wanted to learn whether training on PPE usage and volunteering (e.g., healthcare settings) during the pandemic influence students' perceived risk and knowledge. Medical students were recruited to support healthcare workers during the initial stages of the pandemic when hospitals were inundated with COVID-19 cases. This occurred not only in Italy but also in other countries (6–9). Importantly, the study highlights the crucial role of infection disease prevention and PPE usage, both of which can influence medical students' safety. The recent literature suggests that medical students may not receive adequate training in infectious disease prevention (10–12). Thus, our study adds to literature by examining the role of medical students training along with other factors that may influence their ability to handle public health emergencies (PHE).

## Method

### Study Design, Participants, and Setting

The study involved an anonymous cross-sectional survey of medical students attending a 6-year program at the School of Medicine and Surgery, University of Bologna. The Medical School administered the survey on an e-learning platform from May 18 to 31, 2020. The Dean of Medical School sent an email invitation to all the enrolled students to participate in the survey. Students accessed the platform using their personal university email account. This strategy prevented multiple entries from the same individual. The university's Bioethics Committee approved the study on May 11, 2020.

### Survey Instrument

A group of advanced medical students and residents attending the Hygiene and Preventive Medicine Program developed the survey using the Delphi method coupled with an extensive literature review. Survey questions were adapted and translated to Italian from a World Health Organization instrument assessing knowledge, risk perceptions, preventive behaviors and trust regarding the COVID-19 pandemic (13).

The questionnaire used included six domains: (1) Students' characteristics, (2) Adoption of preventive measures, (3) Trust in information sources, (4) Training received and knowledge of PPE usage, and (5) Perceived risk of infection (PRoI). A sixth domain included concerns and opinions about the pandemic; however, it had little relevance to the study goals and therefore was not considered.

Demographic characteristics included: age, gender, academic year, and whether students had participated in volunteer activities during the pandemic. Adoption of preventive measures included nine dichotomously scored items assessing adoption of evidence-based preventive measures (e.g., avoid touching facial parts, self-isolation, and wearing facemasks). We formed a unit-weighted index based on the nine measures with higher scores indicating greater adherence to preventive behaviors.

Trust in the source of information included 11 items assessing students' level of trustworthiness of different sources of information regarding COVID-19. Each item was rated using a 5-point Likert scale ranging from “very little” to “very much,” with individuals who did not select a source assigned “0.” Sources of information were grouped based on traditional media (i.e., TV, press, and radio), web-based media (i.e., web search engines, YouTube, and social media) and scientific sources (i.e., institutional websites, scientific journals, pre-print publications, and medical consultation). We formed an average level of trust within each information source group.

Training on biological risk prevention included two items assessing education received about biological risk and PPE usage. These two measures were combined into two dummy coded measures contrasting “no training” vs. “biological risk training” vs. “biological risk and PPE usage training.” A 5-point scale assessed the need for additional training on PPE usage with responses ranging from “not needed” to “definitely needed.” Effectiveness of four types of instructional strategies was assessed with a 10-point response format ranging from “ineffective” to “extremely effective.” The latter two questions are used only for descriptive purposes. Knowledge of using PPE was measured by a unit-weighted index calculated as the sum of seven dichotomously scored items assessing the number of PPE the student was able to use (e.g., surgical mask, medical gloves, single-use gown), with higher scores indicating greater knowledge.

Three questions assessed PRoI from SARS-CoV-2 in different activities: routine daily activities, educational activities in an academic setting, and educational activities in a healthcare setting. All three questions used the same 10-point response format ranging from “no risk” to “high risk.”

The Supplementary Materials includes an English-translated version of the survey questions included in this paper. Only students enrolled in the third or subsequent years answered questions on training, knowledge of using PPE, and risk perception in a healthcare setting, as clinical rotations commence during the third year of medical school.

### Statistical Analysis

We compared the representativeness of the sample with respect to the total medical student population based on demographic measures. Continuous variables were summarized using mean, standard deviation (±SD), range, median and inter-quartile range [IQ range]. Categorical variables were reported as absolute and percentage frequencies. We examined histogram plots, results of the Shapiro-Wilk test as well as skewness for evidence of distributional normality and selected appropriate parametric or non-parametric tests for analysis of continuous variables. Paired-sample *t*-tests compared students' perceived risk across different settings. Wilcoxon signed-rank test compared students' trust in different information sources, given the underlying ordinal distribution of these measures. The Bonferroni correction adjusted for multiple comparisons. We used one-way analysis of variance and independent-sample *t*-tests to compare perceived risk between different groups of students. Results were reported as mean and SD as well as percentage differences. Levene's *F*-test examined the between-groups equal variances assumption. The Wilcoxon Mann-Whitney and Kruskal-Wallis tests were used to examine the association between categorical variables and knowledge of PPE usage. We used the Pearson's correlation coefficient to examine the association between normally distributed variables, and the Spearman correlation coefficient to examine the association between count and ordinal variables.

We used path analysis to examine the multivariate relations between gender, age, volunteer status, trust in information sources, adoption of preventive measures, training on biological risk and PPE usage, and PRoI. The model posits associations between exogenous and endogenous measures (perceived risks), correlations among the exogenous measures, and residual associations among the endogenous measures, net of the effect of the predictors. Model results include standardized regression coefficients with 95% confidence intervals (95% CIs). We report model estimates based on both the fully saturated model and a trimmed model not including less significant paths. This decision coincides with recent discussions in the literature regarding the importance of distinguishing between statistical and clinical significance, including using *p*-values as a continuous measure to avoid eliminating clinically meaningful effects merely based on the 0.05 cutoff (14, 15). The model's goodness of fit was assessed using the Comparative Fit Index (CFI) (16), Tucker Lewis Index (TLI) (17), Root Mean Square Error Approximation (RMSEA) (18) and Standardized Root Mean Residual (SRMR) (19). A well-fitting model should have CFI and TLI > 0.95 and both RMSEA and SRMR <0.05. We examined the effects of the exogenous measures on knowledge of PPE usage (count variable) using a multivariate Poisson regression model. We reported model results as incidence rate ratios (IRRs) with 95% CIs. An IRR >1 indicates an increase in the probability of having one more unit of the outcome (i.e., greater knowledge of using PPE). We excluded the variable academic year from the multivariate analyses to avoid collinearity with age. *Post-hoc* power analysis indicated that the sample size was adequate to detect significant differences on perceived risk between subgroups. A Monte Carlo simulation also reinforced the study was adequately powered (>90%) for the multivariate path analysis. Statistical analyses were carried out using Stata statistical software version 15 and Mplus software version 8.6 (20, 21).

## Results

### Students' Characteristics and Sample Representativeness

Six hundred and fifty-five medical students took the online survey. Analyses included 537 (82%) respondents who provided complete data. Table 1 describes the sample characteristics. The sample mean age was 23.4 years (range: 19–39) and 61.3% (*n* = 329) of respondents were female. The percentage of students in each academic year varied between 8.4% (2nd year) and 27.2% (6th year) with 439 (81.8%) students enrolled in the third or subsequent years. Compared to the overall medical student population (*N* = 2147), the survey sample overrepresented students enrolled in the third and subsequent years (81.8 vs. 67.5% in the reference population, *p* < 0.001) and females (61.3 vs. 55.5% in the reference population, *p* = 0.007). The sample of medical students and the overall student population had similar age distributions, both with a mean of 23 years (range 19–39). On average, students used seven different sources of information and all of them used at least two. Students reported that they trusted mostly scientific sources (4.1 ± 1.0) compared to the other sources of information (traditional media, 2.6 ± 1.1, web-based media, 2.3 ± 1.0, all p's < 0.001). However, scientific journals ranked only sixth in the frequency of use (Supplementary Figure 1A). Conversely, social media websites were more frequently used (79.2%) but reported as the least trustworthy source (Supplementary Figure 1B). On average, students reported to have adopted eight out of nine evidence-based preventive measures (52% females vs. only 32% males adopted all nine measures). Among students enrolled in the third or subsequent years, 45% reported they had received training regarding biological risk and use of PPE, 39% reported they had received training on biological risk only. The remaining 16% reported having received no training on either risks or PPE use at all. Ninety-one percent (*n* = 401) of students requested additional training on the use of PPE. Students regarded peer-to-peer activities and training with a tutor as the most effective instructional approaches (7.5 ± 2.5, 8.5 ± 2.0, respectively). On average, students were able to use four out of seven types of PPE, with little knowledge on the use of gowns (26.2%, *n* = 115) and facial shields (14.1%, *n* = 62). Paired *t*-tests indicated that the PRoI was on average higher for activities performed in the hospital setting (7.1 ± 1.9) as compared to perceived risk for activities in the academic setting (6.1 ± 2.0) and activities in daily living (4.1 ± 1.8) (all p's < 0.001).

**Table 1:** Medical students sample characteristics.

	***N* = 537**
**Gender, n (%)**	
Male	208 (38.7)
Female	329 (61.3)
**Age, mean** **±** **SD**	23.4 ± 3.1
**Academic year, n (%)**	
1st year	53 (9.9)
2nd year	45 (8.4)
3rd year	116 (21.6)
4th year	74 (13.8)
5th year	103 (19.2)
6th year	146 (27.2)
**Volunteer activity, n (%)**	
No	453 (84.4)
Yes	84 (15.6)
**Number of information sources used, median [IQ range]**	7 [6–8]
**Trust in information sources, mean** **±** **SD**	
Scientific sources	4.1 ± 1.0
Traditional media	2.6 ± 1.1
Web-based media	2.3 ± 1.0
Number of preventive measures adopted, median [IQ range]	8 [7–9]
**Training on biological risk and PPE usage** ^**a**^ **, n (%)**	
None	70 (16.0)
Biological risk only	170 (38.7)
Biological risk and PPE usage	199 (45.3)
**Need for additional training on PPE usage** ^**a**^ **, n (%)**	401 (91.3)
**Effectiveness of instructional method**^**a**^**, mean** **±** **SD**	
Online lessons	6.9 ± 2.5
Informative material	6.7 ± 2.4
Peer-to-peer training	7.5 ± 2.5
Training with tutor	8.5 ± 2.0
**Perceived risk, mean** **±** **SD**	
Daily living activities	4.1 ± 1.8
Academic activities	6.1 ± 2.0
Healthcare activities^a^	7.1 ± 1.9
**Knowledge of PPE usage (number of PPE able to use)** ^**a**^ **, median [IQ range]**	4 [2–5]

a *Evaluated among students enrolled at third or subsequent years (n = 439)*.

### Characteristics Associated With Perceived Risk of Infection and Knowledge of PPE Usage

Supplementary Table S1 contains the bivariate associations between the exogenous variables and the outcome measures of PRoI and knowledge of PPE usage. Age, adoption of preventive measures, and trust in information sources were positively associated with perceived risk in one or more settings. Compared to males, females reported a 9-point higher percentage in PRoI in the academic setting (6.3 ± 1.9 vs. 5.8 ± 2.0, *p* = 0.002). Moreover, risk perception differed between students participating in volunteer work compared to those who did not. Those who participated in volunteer work reported 16-point higher percentage in perceived risk for daily activities (4.6 ± 1.7 vs. 4.0 ± 1.8, *p* = 0.004) and 5-point lower percentage in perceived risk for in-hospital activities (6.8 ± 1.7 vs. 7.2 ± 1.9, *p* = 0.099) compared to those who did not volunteer. Perceived risk of infection during educational activities was lower for those who received training on PPE use compared to those who did not (−10 and −9% in perceived risk in academic and healthcare activities, respectively). With respect to knowledge on how to use PPE, we found a positive association with age, male gender, volunteer activity and training received (Supplementary Table S1).

### Multivariate Path Model and Poisson Regression Model

**Figure 1:**
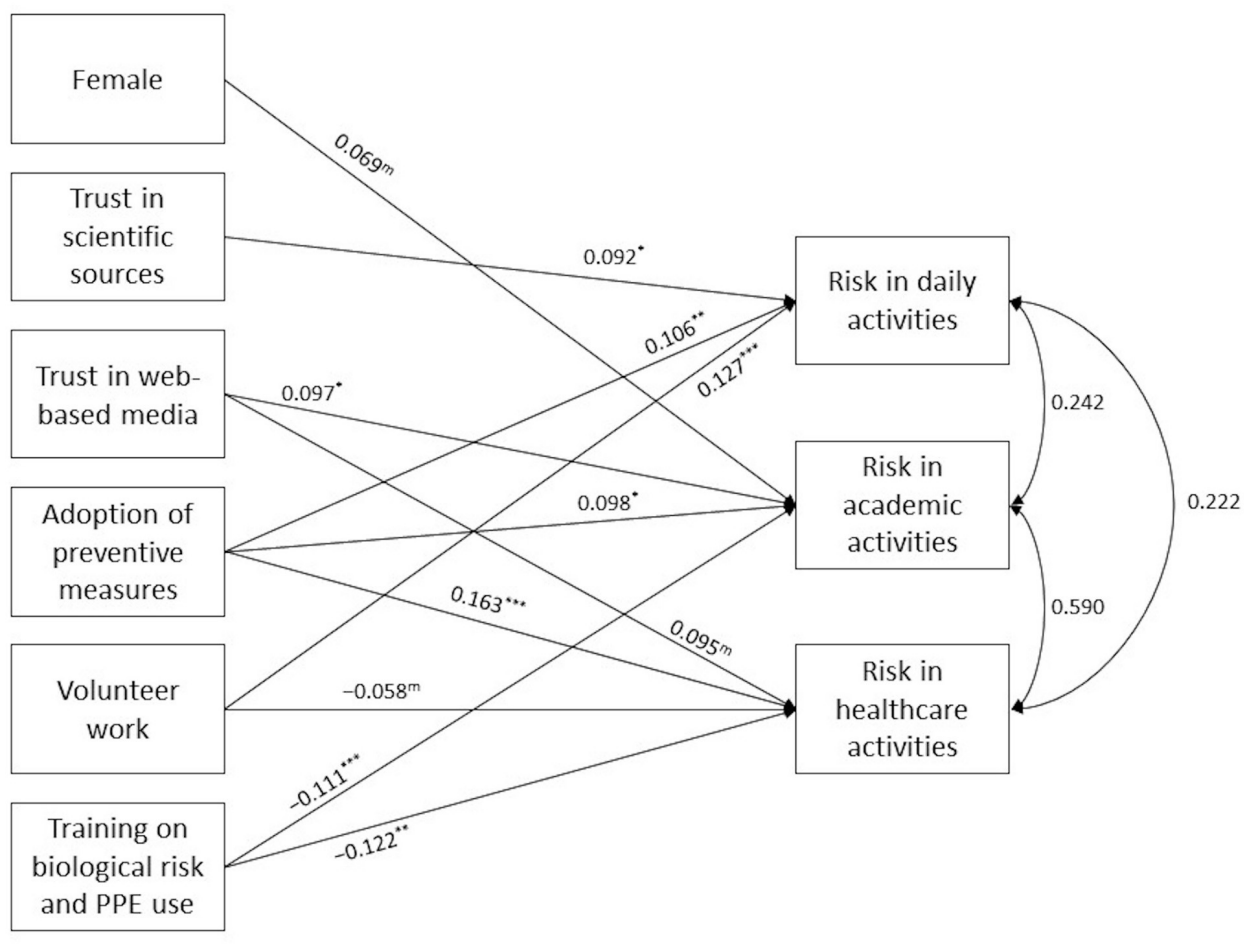
Path diagram depicting estimates from the trimmed path model. Single-headed arrows indicate the relationship between exogenous and endogenous variables. The number reported over each single-headed arrow represents the standardized regression coefficient. Double-headed arrows and the values reported near them indicate the correlation between the edogenous variables.

Table 2 reports the full set of estimates with 95% CIs from the multivariate path analysis. Table 2 should be read in conjunction with Supplementary Table S2 containing the estimated correlations among the exogenous variables from the full model. Figure 1 includes the results of a model trimmed of less significant paths. Based on the various benchmark fit indices, the trimmed model provided a good fit to the sample data: $\chi_{\left(16\right)}^{2}$ = 17.65, *p* = 0.345; CFI = 0.997; TLI = 0.991; RMSEA = 0.014; SRMR = 0.021. As depicted in Figure 1, female gender was associated with PRoI during academic activities. There was a positive association between the number of preventive practices adopted and the PRoI in all the three settings. This relation was larger in magnitude with perceived risk in healthcare (β = 0.163, 95%CI 0.078–0.247). In addition, training on biological risk and PPE use was negatively associated with PRoI during educational activities in both academic (β = −0.111, 95%CI −0.790– −0.085) and healthcare setting (β = −0.122, 95%CI −0.806– −0.123). Moreover, volunteer status was positively associated with perceived risk in daily living activities (β = 0.127, 95%CI 0.049–0.205) and to a lesser degree negatively associated with perceived risk in healthcare (β = −0.058, 95%CI −0.123–0.008). Compared to the fully saturated model, in the trimmed model the positive effect of both trust in scientific sources and trust in web-based media on at least one of the three perceived risk outcomes was larger in magnitude than the effect of traditional media. Similarly, in the trimmed model the positive effect of age diminished in significance.

**Table 2:** Fully saturated path model estimates.

	**Perceived risk in daily**			**Perceived risk educational**			**Perceived risk educational**		
	**living activities**			**activities in academic setting**			**activities in healthcare**		
	**β^a^**	**95% CI**	***p*-value**	**β**	**95% CI**	***p*-value**	**β**	**95% CI**	***p*-value**
Age	0.048	−0.042 – 0.138	0.296	0.092	0.003 – 0.182	0.039	0.089	0.003 – 0.176	0.039
Female gender	0.026	−0.060 – 0.112	0.554	0.098	0.013 – 0.183	0.025	0.049	−0.041 – 0.139	0.284
Volunteer activity	0.123	0.044 – 0.203	0.002	−0.028	−0.103 – 0.046	0.452	−0.070	−0.145 – 0.004	0.064
Training on biological risk only	−0.021	−0.165 – 0.123	0.774	−0.019	−0.141 – 0.104	0.767	−0.013	−0.136 – 0.109	0.829
Training on biological risk and PPE usage	−0.076	−0.222 – 0.069	0.305	−0.135	−0.262 – −0.007	0.039	−0.135	−0.263 – −0.007	0.040
Adoption of preventive measures	0.091	0.012 – 0.170	0.024	0.086	0.009 – 0.164	0.029	0.155	0.070 – 0.239	<0.001
Trust in scientific sources	0.078	−0.004 – 0.159	0.060	−0.036	−0.129 – 0.057	0.449	−0.064	−0.143 – 0.016	0.117
Trust in traditional media	0.092	0.005 – 0.179	0.038	0.083	−0.005 – 0.171	0.065	0.016	−0.078 – 0.110	0.740
Trust in web-based media	−0.007	−0.096 – 0.081	0.872	0.077	−0.011 – 0.166	0.089	0.087	−0.013 – 0.187	0.088

a *Regression coefficients (β) are standardized; 95% CI indicates 95% confidence interval*.

In the Poisson regression model, age, gender, volunteer activity and training received were more significantly associated with knowledge on the use of PPEs as compared to the other factors (Table 3). Students who volunteered and those having received training on PPE usage were able to use more types of PPE compared to the reference group. Females had lower probability of knowing how to use PPE as compared to males. Moreover, knowledge of PPE usage increased with age.

**Table 3:** Association between independent variables and knowledge on PPE usage: results from multivariate Poisson regression model.

	**IRR^**a**^**	**95% CI**	***p*-value**
Age	1.03	1.02–1.05	<0.001
Female gender	0.89	0.80–0.99	0.038
Volunteer activity	1.20	1.06–1.36	0.005
Training			
None	1.00	–	–
Biological risk only	1.10	0.93–1.29	0.274
Biological risk and PPE usage	1.39	1.19–1.63	<0.001
Adoption of preventive measures	1.04	0.99–1.09	0.136
Trust in scientific sources	1.01	0.96–1.06	0.623
Trust in traditional media	1.00	0.95–1.05	0.993
Trust in web-based media	1.02	0.97–1.08	0.434

a *IRR indicates incidence rate ratio; 95% CI indicates 95% confidence interval*.

## Discussion

During the early stages of the pandemic Italian medical students were recruited to support the healthcare fight against COVID-19. As a result, it becomes important to examine students' PRoI and more specifically what factors influence risk that may be amenable to educational intervention. The findings of this study provides initial evidence of the role of information trust, adoption of prevention measures, training, and volunteering on both PRoI and knowledge of PPE usage. Each one of these findings is discussed accordingly.

In terms of preferred sources of information, students favored online sources and social media, consistent with previous studies (22, 23). Medical students reported they had greater trust in scientific publications compared to other sources of information. However, scientific journals were not in the top-ranked sources used. This gap may arise from lack of access, lack of understanding of scientific literature, language barriers, and lack of knowledge how to use university libraries (24). Knowledge and ability to consult online libraries and scientific literature web search engines are skills that could be easily incorporated into the medical school curriculum (25, 26). A previous study (27) showed that medical students share information with their families and friends. The same students also rely heavily on social media to share their medical knowledge. As a result, students can play an important role in promoting health, for instance during a pandemic, to the general population. This will help to offset factors that precipitate infodemics, which that can produce misleading health information.

The adoption of preventive measures contained multiple items assessing standard practices that medical providers should be aware of on a day-to-day basis. Medical students in our sample, especially females, reported they adopted most of the recommended preventive measures as part of their everyday life during the pandemic, a finding in line with other studies (22, 28, 29). With respect to training, a minority of students indicated they were sufficiently educated in infectious disease prevention and PPE usage. Consistent with other studies, students also indicated there is an urgent need for additional instruction and continued training on PPE use (30, 31). Furthermore, students preferred peer-to-peer education and mentoring approaches compared to more traditional educational methods. The current findings are consistent with other recent studies demonstrating the lack of practical experience of PPE usage (10, 11, 30, 31). Effective use of personal protective equipment (PPE) is essential to protect personnel and patients in healthcare settings (11).

Overall, students reported moderate levels of perceived risk of infection; this risk was higher for attending clinical training compared to academic or daily living activities. Student also reported a lack of essential skills for using PPE, being able to use on average only four out of seven different types of protective equipment. When set in the multivariate framework we obtained a clearer picture of how all of the exogenous factors relate not only to each other but also to PRoI. Higher trust in information sources related differently to higher perceived risks. Trust in scientific sources was associated with higher risk only in daily activities whereas trust in web-based media was associated with risk in both academic and healthcare activities. One possible reason for these differences may involve the different appeal that each media source has on different people as their tastes in information sources may vary considerably. Adoption of preventive measures was associated with higher perceived risk in all three settings. Moreover, the largest effect overall in the model was between adoption of preventive measures and perceived risk in healthcare activities. The positive relationship could potentially indicate that medical students at risk for viral infection respond by implementing preventive practices. The non-recursive nature of this relationship in a cross-sectional survey can also mean these relations are reciprocal and perceived risk precedes adoption of preventive measures.

The path model also revealed that training on both biological risk and use of PPE was associated with less perceived risk both in the hospital setting and in academic activities and to a lesser extent daily activity. This difference in the effect of training arises perhaps because medical students are less concerned about viral transmission when going about their daily business. Moreover, the training on PPE usage may have facilitated the development of skills that reduce their PRoI. Because medical students providing care to patients are at high risk for contracting as well as transmitting COVID-19 (28), training on biological risk and PPE usage could serve to reduce virus spread. Notably, the early stages of the pandemic particularly affected healthcare workers, with 305 deaths due to COVID-19 among Italian healthcare workers, as of January 31, 2021 (32). One explanation for this alarming figure is perhaps a lack of access and ability to use PPE (33). In our study, the multivariate Poisson regression model confirmed that knowledge of PPE usage was directly associated with the training medical students received. This finding highlights that one remedy to the lack of knowledge and ability to use PPE includes extending specific training on PPE usage to medical students early in their education and especially during their clinical rotations, when they encounter patients and spend considerable time at the hospital.

Volunteering during the pandemic was associated with a higher perceived risk of contracting COVID-19 in daily living activities and a lower perceived risk of contracting COVID-19 in a hospital setting. These findings could perhaps indicate that volunteer experiences coupled with training in PPE usage helps medical students understand the risk of viral infection. The same training that they receive in the hospital may not mitigate the risk of infection in the real world where they perceived to have less control. Results from the Poisson model also reinforce the beneficial effect of volunteering on students' knowledge about PPE usage. During the COVID-19 pandemic, many student-driven initiatives and volunteer efforts were reported worldwide, highlighting benefits for students and healthcare services (6–9, 34, 35). Combined, these results collectively show that volunteer activities should be encouraged by educational institutions.

Compared to males, females reported higher risk perception. Females also reported adopting a greater number of preventive measures, but, at the same time they reported having less knowledge of PPE usage. These results are in line with other studies (10, 36) and perhaps suggest that females may not feel as adequately prepared for dealing with the pandemic as males, moreover this gap in their knowledge exacerbates their perception of risk.

Finally, age related positively to perceived risk, especially in academic and healthcare activities. Moreover, age was also positively associated with knowledge of PPE usage. Given the nature of the medical curricula for Italian students, most of the older students were obtaining hands-on training during clinical rotation as opposed to classroom-based education. As a result, they are more aware of the need for infectious disease prevention, a finding in line with other studies (29).

There are several limitations associated with this study worth noting. First, only Italian medical students comprised the sample and their training may be unique compared to other programs. However, worldwide medical students were recruited to assist in healthcare settings to address the pandemic. Therefore, our findings may have relevance for different healthcare settings as well as different countries. Second, the sample may have some selection bias because students voluntarily participated in the survey. Indeed, the sample overrepresented students attending the latter years of medical school. Their advanced state of medical education and interest in clinical rotations could have also potentially influenced their participation and responses. Moreover, there was a limited timeframe for data collection (<2 weeks) suggesting limited coverage. Third, the cross-sectional design does not allow us to determine causality between training and/or volunteer work and the outcome measures. Fourth, the associations found could be spurious because of other non-explored factors, e.g., variables that measure personality traits, all of which can influence perceived risk and knowledge. Despite these limitations, we believe our results highlight important facets of medical school education; ones that should be addressed, especially during critical periods when healthcare is in high demand such as during the pandemic.

Efforts are already under way to translate the results of this survey and implement them into practice. For example, PPE training is now available for all medical students at the University of Bologna in order to increase their knowledge of and skills to deal with the virus. This training was mandatory for the students returning to clinical rotations. Furthermore, medical students were engaged in public health outreach efforts, they developed brochures and infographics about personal protective measures addressed to patients, their families, and surrounding communities. These materials are available in different languages (Italian, English, Chinese, and Arabic) and disseminated to diverse communities to increase awareness about protective measures, in particular in hard-to-reach populations (i.e., homeless and migrants).

The present study adds to the growing literature on medical students' education during the pandemic. An important distinction that comes from this study is that adoption of preventive measures and trust in information sources are efficient predictors of perceived risk but not knowledge whereas training and volunteering work affect both outcomes. Training and volunteer activities influence students' effort to counteract the COVID-19 pandemic. Taken as a whole, this suggests that there is a continued need for implementing training strategies targeting medical students' education in public health measures, use of personal protective equipment that reduces their exposure to biological risk transmission, and ensuring this training commences at the earliest part of their medical education. In addition, our findings highlight the role of students' engagement in voluntary work to enhance hospital capacity and as a means for professional development. Medical students will become future medical professionals, thus a specific training responding to contemporary challenges is fundamental to support the healthcare system's ability to address pandemic situations that may arise in the future.

## Data Availability Statement

The raw data supporting the conclusions of this article will be made available by corresponding author on reasonable request (research purpose).

## Ethics Statement

The studies involving human participants were reviewed and approved by Bologna University Bioethics Committee (https://www.unibo.it/en/research/research-facilities/ethics-committees/bioethics-committee). The patients/participants provided their written informed consent to participate in this study.

## Author Contributions

MG, FR, MPF, and DG contributed to conception and design of the study. MG, FR, and DT collected the data. MG and EM organized the database and wrote the first draft of the manuscript. EM performed the statistical analysis. FR and CR helped in writing sections of the first manuscript draft. MPF, ES, and DG supervised and edited the first manuscript draft. EM, MG, and LS revised the manuscript in depth after first-cycle revisions. All authors read and approved the submitted version.

## Conflict of Interest

The authors declare that the research was conducted in the absence of any commercial or financial relationships that could be construed as a potential conflict of interest.

## Publisher's Note

All claims expressed in this article are solely those of the authors and do not necessarily represent those of their affiliated organizations, or those of the publisher, the editors and the reviewers. Any product that may be evaluated in this article, or claim that may be made by its manufacturer, is not guaranteed or endorsed by the publisher.
