# Supplementary material for: Assessing the Role of Trust in Information Sources, Adoption of Preventive Practices, Volunteering and Degree of Training on Biological Risk Prevention, on Perceived Risk of Infection and Usage of Personal Protective Equipment Among Italian Medical Students During the SARS-CoV-2 Pandemic
Source: Front Public Health. 2021 Oct 28;9:746387. doi: 10.3389/fpubh.2021.746387 (PMC8581255; doi:10.3389/fpubh.2021.746387)
Supplement: Supplementary file 1 [file Data_Sheet_1.PDF]

Supplementary Material

Survey questionnaire

INDIVIDUAL CHARACTERISTICS

1. How old are you? (in years)

2. What is your gender?
   - Female
   - Male
   - Other/prefer not to specify

3. What is your current year in Medical School?
   - First
   - Second
   - Third
   - Fourth
   - Fifth
   - Six

4. Did you volunteer in relation to COVID-19 emergency during the first months of the pandemic?
   - No
   - Yes

ADOPTION OF PREVENTIVE MEASURES

5. Which of these measures have you taken to prevent novel coronavirus infection?

	Yes	No
Avoiding touching my eyes, nose and mouth		
Cleaning hands with soap and water or alcohol-based hand rub		
Staying home if I do not feel well or if I have cold		
Self-isolation		
Covering mouth and nose with bent elbow or tissue when coughing or sneezing		
Wearing face mask		
Physical distancing (interpersonal distance of at least one metre)		
Disinfection of surfaces		
Disinfection of my mobile phone		

INFORMATION SOURCES

6. Which sources did you use to get information about COVID-19 emergency? Assign a trustworthiness score from 1 (very little) to 5 (very much) rate each of the sources you used:
- Television
- Radio
- Press
- Web search engine
- Institutional websites
- YouTube
- Social media (Facebook, Twitter, Instagram…)
- Medical consultation
- Relatives and friends
- Scientific Journals
- Pre-print publications

PERCEIVED RISK OF INFECTION

7. On a scale of 1 (no risk) to 10 (highest risk), what do you think your SARS-CoV-2 infection risk is during your activities of daily living (in the next 30 days)?

8. On a scale of 1 (no risk) to 10 (highest risk), what do you think your SARS-CoV-2 infection risk is during your educational activities in the academic setting (in the next 3 months)?

9. On a scale of 1 (no risk) to 10 (highest risk), what do you think your SARS-CoV-2 infection risk is during your educational activities in the healthcare setting (in the next 3 months)?

TRAINING RECEIVED and KNOWLEDGE OF PPE USAGE

10. During your university education, have you ever received information on biological risk prevention?
- Yes
- No
- I don’t remember

11. During your university education, have you ever received information on PPE usage?
- Yes
- No
- I don’t remember

12. Considering the resumption of in-presence educational activities, do you think further training on PPEs usage is needed?
1. No, it is not needed
2. It is probably not needed
3. I don’t know
4. It is probably needed
5. Yes, it is definitely needed

13. On a scale of 1 (ineffective) to 10 (highly effective), how much do you think each of the following training method is effective?

- Online lessons
- Informative material
- Training with tutor
- Peer-to-peer training

14. Based on your current knowledge, do you know how to don the following PPEs?

	No	Yes	I don't know
Surgical mask			
Medical gloves			
Surgical cap			
Surgical shoes			
Single-use gown			
Respirator mask (FFP2/FFP3)			
Facial shield			

15. Based on your current knowledge, do you know how to doff the following PPEs?

	No	Yes	I don't know
Surgical mask			
Medical gloves			
Surgical cap			
Surgical shoes			
Single-use gown			
Respirator mask (FFP2/FFP3)			
Facial shield			

Supplementary Figures and Tables

Supplementary Figure S1. Students' use and trustworthiness of different type of information sources. (A) Students' use of different type of information sources. (B) Mean trustworthiness score assigned by students to each type of information source, bars represent the standard deviation.

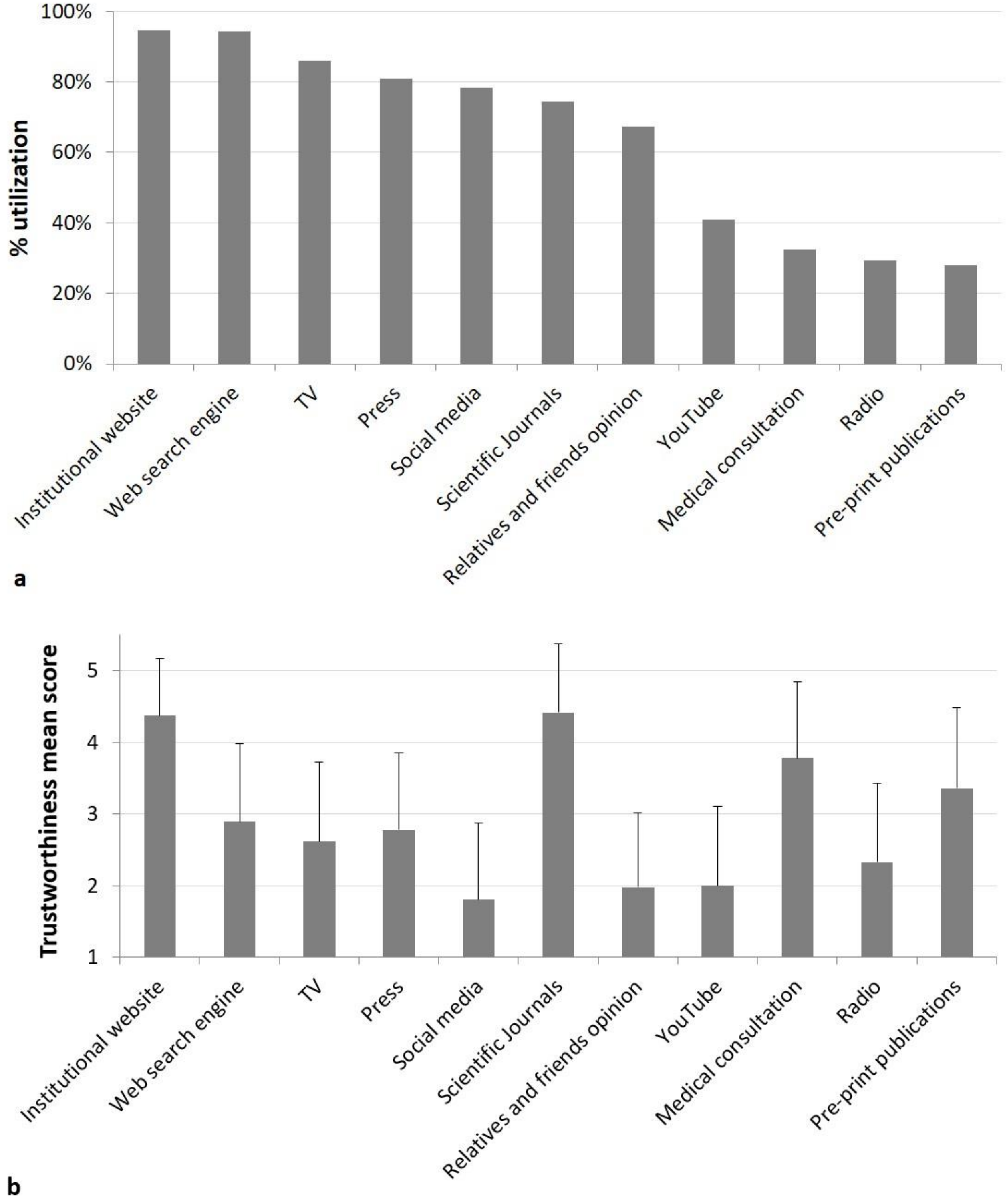

Supplementary Table S1. Association between independent (column) and dependent (top row) variables.

	Perceived risk daily living activities	p-value	Perceived risk educational activities in academic setting	p-value	Perceived risk educational activities in healthcare	p-value	Knowledge of PPE usage	p-value
Age	0.043	0.324[1]	0.093	0.032[1]	0.117	0.014[1]	0.208	<0.001[2]
Gender		0.315[3]		0.002[3]		0.110[3]		0.046[4]
Male	4.0±1.8[3]		5.8 ±2.0		6.9±1.9		4 [2 – 5][4]	
Female	4.1±1.8		6.3 ±1.9		7.2±1.9		3 [2 – 5]	
Academic year	0.070	0.107[2]	0.060	0.165[2]	0.113	0.018[2]	0.228	<0.001[2]
Volunteer activity		0.004[3]		0.434[3]		0.099[3]		0.006[4]
No	4.0±1.8		6.2 ±2.0		7.2±1.9		3 [2 – 5]	
Yes	4.6±1.7		6.0 ±1.8		6.8±1.7		4 [2 – 5]	
Training		0.427[5]		0.016[5]		0.011[5]		<0.001[6]
None	4.3 ±1.9		6.5 ±1.8		7.5 ±1.7		3 [1 – 4]	
Biological risk only	4.2 ±1.7		6.3 ±1.8		7.3 ±1.8		3 [2 – 5]	
Biological risk and PPE usage	4.0 ±1.9		5.9 ±2.1		6.8 ±2.0		4 [2 – 5]	
Adoption of preventive measures	0.113	0.009[2]	0.123	0.004[2]	0.144	0.003[2]	0.065	0.177[2]
Trust in scientific sources	0.035	0.416[2]	-0.045	0.301[2]	-0.040	0.399[2]	0.021	0.657[2]
Trust in traditional media	0.062	0.152[2]	0.085	0.049[2]	0.022	0.642[2]	0.005	0.913[2]
Trust in web-based media	0.032	0.466[2]	0.103	0.017[2]	0.078	0.103[2]	-0.003	0.959[2]

Notes: [1] Pearson correlation coefficient assessed the association between continuous normally distributed variables; [2] Spearman rank correlation assessed the association between count and/or ordinal variables; [3] Student's t-test compared the mean perceived risk scores among levels of categorical variables (2 categories); [4] Non-parametric Mann-Whitney test compared knowledge of PPE usage median [IQ range] scores among levels of categorical variables; [5] One-way ANOVA compared mean perceived risk scores among levels of training (>2 categories); [6] Non-parametric Kruskal Wallis test compared knowledge of PPE usage median [IQ range] scores among levels of training (>2 categories).

Supplementary Table S2. Path model estimated correlations.

	Age	Gender	Volunteer activity	Training on biological risk only	Training on biological risk and PPE usage	Trust in scientific sources	Trust in traditional media	Trust in web-based media	Adoption of preventive measures
Age	1.00								
Gender	-0.049	1.00							
Volunteer activity	0.055	-0.089	1.00						
Training on biological risk only	-0.056	0.031	-0.051	1.00					
Training on biological risk and PPE usage	-0.091	-0.037	0.000	-0.723	1.00				
Trust in scientific sources	-0.050	0.057	0.011	0.020	0.013	1.00			
Trust in traditional media	-0.165	0.044	-0.046	-0.034	0.074	0.051	1.00		
Trust in web-based media	0.064	0.118	-0.021	-0.010	-0.033	0.017	0.167	1.00	
Adoption of preventive measures	0.004	0.230	0.034	0.002	-0.016	0.081	0.102	0.042	1.00
